# Gender, Populism, and the QAnon Conspiracy Movement

**DOI:** 10.3389/fsoc.2020.615727

**Published:** 2021-01-21

**Authors:** Lorna Bracewell

**Affiliations:** Flagler College, St. Augustine, FL, United States

**Keywords:** populism, gender, conspiracy, Trumpism, Andrea Dworkin, femininity, QAnon

## Abstract

According to one recent review of the burgeoning interdisciplinary scholarly literature on populism, populism’s “relationship with gender issues remains largely understudied” (Abi-Hassan, 2017, 426–427). Of those scholarly treatments that do exist, the lion’s share focus on the role of men and masculinity in populist movements. In this essay, I argue scholarly reflection on the relationship of gender and populism should not be limited to this narrow frame. Through a close examination of the complex gender politics of QAnon, a pro-Trump conspiracy movement that burst into the mainstream of U.S. politics and culture with the onset of the global Coronavirus pandemic, I demonstrate that populist deployments of femininity are as rich, complex, and potent as their deployments of masculinity. QAnon, I argue, is a case study in how femininity, particularly feminine identities centered on motherhood and maternal duty, can be mobilized to engage women in populist political projects. Until scholars of populism start asking Cynthia Enloe’s famous question, “Where are the women?,” in a sustained and rigorous way, phenomena that are integral to populism’s functioning will elude us and our understanding of the relationship between gender and populism will remain partial and incomplete (Enloe, 2014).

According to one recent review of the burgeoning interdisciplinary scholarly literature on populism, populism’s “relationship with gender issues remains largely understudied” (Abi-Hassan, 2017, 426–427). Of those scholarly treatments that do exist, the lion’s share focus on the role of men and masculinity in populist movements^1^. This emphasis is understandable, in part, for historical reasons. The first political movement to which the term “populist” was applied,^2^ the U.S. People’s Party of the late nineteenth century, figured “the people” in expressly masculine terms as a virile and virtuous yeomanry pitted against a corrupt and effeminate urban elite (Hofstadter, 1955). Contemporary factors doubtlessly also contribute to this privileging of men and masculinity in populism research. One of the most conspicuous populist movements in the world today, Trumpism, is emphatically masculinist, counting among its most zealous adherents exclusively male neo-fascist groups like the Proud Boys. The Hindu nationalist populism of India’s Bharatiya Janata Party (BJP) under the leadership of Prime Minister Narendra Modi also features a heady dose of masculinism, and parallels between Modi and Trump, both would-be authoritarians who intimidate and exclude marginalized communities, attack the free press, and deploy misinformation and propaganda to stoke racial, ethnic, religious, and caste resentments, have often been noted by pundits (Boot, 2019; Tasseer, 2019).

While men and masculinity have figured prominently in populist movements past and present, scholarly reflection on the relationship of gender and populism should not be limited to this narrow frame. Populist deployments of femininity are as rich, complex, and potent as their deployments of masculinity and women are often key players in populist movements. Consider, for example, QAnon, a pro-Trump conspiracy movement that originated in the depths of the anonymous internet imageboard 4chan in 2017 and burst into the mainstream of U.S. politics and culture with the onset of the global Coronavirus pandemic in the spring of 2020. QAnon is a case study in how femininity, particularly feminine identities centered on motherhood and maternal duty, can be deployed to engage women in populist politics.

A broad-strokes summary of the QAnon movement is hard to formulate. QAnon is an umbrella term for a baroque set of conspiracy theories alleging without evidence that the world is controlled by a secret cabal of Satan-worshipping pedophiles who are abducting, abusing, and ritualistically murdering children by the thousands. This global child trafficking ring, QAnon adherents believe, counts among its members powerful elites like Pope Francis and Ellen DeGeneres as well as many prominent members of the Democratic Party like Hillary Clinton and Barack Obama. President Donald Trump also plays a leading role in the QAnon mythos as a secret-agent/warrior/messiah figure. Recruited by top military generals to run for president in 2016, Trump has been working tirelessly behind the scenes ever since to defeat the Satanist cabal. What gives the QAnon movement its unmistakable populist tinge is the role it scripts for its supporters in the apocalyptic confrontation between Trump and the cabal that QAnon devotees believe is imminent. A high-ranking intelligence officer or military official posting anonymously on the internet as “Q,” signaling his Q level security clearance and access to the most closely guarded secrets of the “deep state,” is communicating Trump’s plans to all brave patriots with ears to hear via encoded messages known as “Q drops.” When the time is right, Q will give the signal and the people will rise up and join Trump in one final Armageddon-like showdown against the forces of darkness that QAnon adherents call “the storm.”

For the first two and a half years of its existence, QAnon attracted a devoted but relatively small coterie of followers. However, in the spring of 2020, as the global Coronavirus pandemic forced millions of people all over the world to hunker down at home and made the internet their almost exclusive connection to the outside world, QAnon’s popularity exploded. According to a study reported in *The Wall Street Journal*, membership in 10 of the largest public QAnon Facebook groups grew by nearly 600% from March to July (Seetharaman, 2020). This same study also found that during this same time period the average follower count of some of the largest public Instagram accounts promoting QAnon beliefs more than quadrupled. Other journalists have reported that a significant number of these more recent QAnon converts are women introduced to QAnon ideology through images, videos, and stories shared by some of the most popular beauty, lifestyle, and parenting influencers on Instagram (Breland, 2020; Butler, 2020; Flora, 2020; Kelly, 2020; Tiffany, 2020).

While the juxtaposition of Instagram’s exaggeratedly feminine, pastel-laden aesthetic with messages like “The deep state is evil and Satanic” may seem jarring, it is not surprising that QAnon’s central message—act boldly to save the children from an invisible yet omnipresent evil—would resonate now in virtual spaces to which millions of women turn every day for advice on how to optimize the health and wellbeing of themselves and their families. We are, after all, in the throes of a global Coronavirus pandemic that the U.S. government has failed to adequately suppress or contain^3^. This means that parents of all genders, but especially mothers upon whom a disproportionate share of the burdens of pandemic-era child-rearing have fallen, are unable to satisfactorily perform even the most basic of parental duties: keeping their children safe and healthy. The QAnon movement ministers to the anxiety this inability to live up to their assigned maternal role triggers in women by providing them entrée into an alternative reality in which the Coronavirus is a hoax and the “real” threat to their children’s health and safety, the deep-state cabal, is something they can actually do something about. Whether it is by collaborating with strangers in a QAnon affiliated social media group to decipher the latest “Q-drop” or calling the National Human Trafficking Hotline to report that online retail giant Wayfair is actually a front for the cabal’s global child sex trafficking ring, women engaged in the QAnon movement are able to assert their feminine identities as protectors of the innocent and regain a sense of maternal efficacy in a moment when both of these things have been severely destabilized by the global pandemic (Seitz and Swenson, 2020). As one QAnon adherent at a “Freedom for the Children” rally in London put it, “Saving our children is far more important than a fake pandemic” (Kelly, 2020).

QAnon’s astonishing success in mobilizing women points toward a characteristic shared by virtually all populist movements that makes populism as a political form particularly hospitable to deployments of femininity. As scholars of populism have long observed, one of the hallmarks of populist movements is their pitting of a uniquely virtuous and unfairly disadvantaged “people” against a corrupt and entrenched “elite” (Kaltwasser et al. 2017, 4). In figuring the people as pure, innocent, and vulnerable, populists, whether wittingly or unwittingly, imbue the people with unmistakably feminine characteristics. With the people always already implicitly feminized by the very nature and structure of populist discourse itself, populists are able to target women with the kind of explicitly gendered appeals we see at work in the QAnon movement with relative ease.

Of course, populist invocations of “the pure people” vs. “the corrupt elite” can also be tailored in ways that downplay these gendered connotations in an effort to unify the people by making gender “an almost unnecessary or even irrelevant category” (Abi-Hassan, 2017, 428). This gender-neutral approach can also work to facilitate women’s participation in populist movements by affording them an opportunity to take their place alongside men as members of the undifferentiated mass of the people. The QAnon movement, at times, operates in this register. For example, its most famous slogan—Where We Go One We Go All—embodies just this sort of appeal to an unmarked and genderless “we.” Nevertheless, it is when populist movements appeal to us as specifically gendered beings with deep-set investments in our masculinity and femininity that they are at their most potent. Such gendered appeals are, without a doubt, one of QAnon’s most significant sources of power. Depending on the nature of their gendered investments, supporters can participate in the QAnon movement as either masculine protectors of the republic who swear an oath to defend the Constitution and become “digital soldiers” in Q’s army or as feminine guardians of hearth and home who organize rallies and social media campaigns to protect children from sexual and moral contamination (Sommer, 2020). The multivocality of its gendered appeals is crucial to QAnon’s success.

Nearly a decade before QAnon became a going concern, gender and politics scholars noted the central role women were playing in another U.S.-based right-wing populist movement, the Tea Party (Sparks, 2014; Deckman, 2016; Schreiber, 2016). One of the most remarkable facets of the Tea Party, a reactionary movement that emerged in the early months of Barack Obama’s first presidential term to champion a blend of economic libertarianism, cultural traditionalism, and nationalism, was its overwhelmingly female leadership. 2008 Republican vice-presidential nominee Sarah Palin and former Minnesota Congresswoman Michelle Bachmann were two of the movement’s most prominent national leaders and studies of grassroots Tea Party organizations have suggested that women were similarly dominant at the local level (Lo, 2012; Deckman, 2016). What facilitated women’s unprecedented levels of engagement with Tea Party populism? According to Deckman, Schreiber, and Sparks, gendered (and, as Sparks perceptively notes, also raced) scripts of maternal guardianship were key to galvanizing Tea Party women. By positioning themselves as “mama grizzlies” rearing up to protect their “cubs” from the predations of tax and spend liberals, the “motherhood frame,” as Deckman calls it, allowed Tea Party women to connect longstanding conservative policy priorities like cutting entitlement programs and expanding gun rights to their maternal duty to defend children and families (2016, 3). Sparks further describes how this “motherhood frame” worked to legitimate Tea Party women’s performances of political anger. “Occupying political positions as ‘mama bears worried about their families,’” Sparks explains, “… offers Tea Party women a way to publicly perform anger that simultaneously defeats the usual charges that angry women are irrational and shrill” (Sparks, 2014, 19). The same maternalist tropes that drew women to the Tea Party also draw them to QAnon, a movement that is far less beholden to libertarian ideology but that affords women similar opportunities to embody their “natural” role of maternal guardian.

Delving further back into the history of right-wing populism in the U.S. can yield additional insight into the complex gender dynamics of the QAnon movement. In 1983, radical feminist theorist Andrea Dworkin published *Right Wing Women* (1983), a pioneering study of the considerable appeal of Reagan-era conservatism, particularly its reactionary sexual and gender politics, to women in the United States. Rather than dismissing conservative women as dupes or diagnosing them with false consciousness, Dworkin argues that what attracts women like Phyllis Schlafly and Anita Bryant to overtly masculinist and patriarchal political projects is that these projects speak in concrete and compelling ways to the vulnerability to sexual exploitation and violence that all women experience under conditions of male dominance. As Dworkin puts it, “The Right in the United States today is a social and political movement controlled almost totally by men but built largely on the fear and ignorance of women. The quality of this fear and the pervasiveness of this ignorance are consequences of male sexual domination over women. Every accommodation that women make to this domination, however apparently stupid, self-defeating, or dangerous, is rooted in the urgent need to survive somehow on male terms” (Dworkin, 1983, 34).

What Dworkin’s analysis in *Right Wing Women* can bring to our understanding of gender and populism today is an appreciation of the sophisticated ways in which right-wing populist movements like QAnon speak to a distinctively feminine set of anxieties and fears to mobilize a distinctively feminine species of anger. QAnon neither dismisses women’s fears of sexual violence as unfounded nor encourages women to confront the actual sources of the sexual violence that threatens them and their families. Rather, QAnon provides women with an opportunity “to take the rage and contempt they feel for the men who actually abuse them, those close to them, and project it onto others, those far away, foreign, or different … ” (Dworkin, 1983, 34). As Dworkin memorably observes, “Women cling to irrational hatreds focused particularly on the unfamiliar so that they will not murder their fathers, husbands, sons, brothers, lovers … Because women so displace their rage, they are easily controlled and manipulated haters … The identities of the dangerous outsiders can change over time to meet changing social circumstances … - but the existence of the dangerous outsider always functions for women simultaneously as deception, diversion, pain-killer, and threat” (Dworkin, 1983, 34). Structurally dependent on men and interpolated into conventionally feminine gender roles that make genuine bids to protect themselves and their children from sexual abuse costly, illegitimate, and threatening to their identities as wives and mothers, women, Dworkin reminds us, are especially ripe for right-wing mobilization. Given these insights, is it any surprise that women have proven receptive to QAnon’s calls to “save the children” by empowering Trump, a man who has been credibly accused of sexual assault and misconduct by more than 20 women and openly bragged about his history of “grab[bing women] by the pussy” (New York Times, 2016; Zhou, 2020)?

As this brief essay has attempted to demonstrate, the gender politics of populism involve more than men and masculinity; populist deployments of femininity are also significant and should not be overlooked. Until scholars of populism start asking Cynthia Enloe’s famous question, “Where are the women?,” in a sustained and rigorous way, phenomena that are integral to populism’s functioning will elude us and our understanding of the relationship of gender and populism will remain partial and incomplete (Enloe, 2014).

## Footnotes

1 Important exceptions include Kampwirth (2010), Spierings et al. (2015), and Rousseau (2011).

2 The editors of The Oxford Handbook on Populism note that the first recorded usage of the term “populist” was in U.S. newspapers in 1891 and 1892 with reference to the U.S. People’s Party (Kaltwasser et al., 2017, 3).

3 As I write this, the U.S. leads the world in Coronavirus deaths and approaches the grim milestone of 300,000 dead from Covid-19.
